# Correlation between tumor location and survival in stage I lung adenocarcinoma and squamous cell carcinoma: a SEER-based study

**DOI:** 10.7150/jca.52572

**Published:** 2021-06-22

**Authors:** Junjie Hu, Mengfan Qi, Xinsheng Zhu, Yan Chen, Jie Dai, Jing Zhang, Gening Jiang, Zhonghong Zhang, Peng Zhang

**Affiliations:** 1 Department of Thoracic Surgery, Shanghai Pulmonary Hospital, Tongji University School of Medicine, Shanghai 200433, China.; 2 Respiration Department II, The First Affiliated Hospital of Shihezi University Medical College, Shihezi, Xinjiang 832000, China.

**Keywords:** location, NSCLC, survival, early stage, chemotherapy

## Abstract

**Background:** Whether location mattered remained controversial in early-stage non-small cell lung cancer.

**Methods:** We conducted a retrospective study with the Surveillance, Epidemiology, and End Results (SEER) database. Overall survival (OS) and lung cancer-specific survival (LCSS) with landmark analysis and restricted mean survival time (RMST) were compared between patients with a tumor in upper lobe and non-upper lobe. The multivariable Cox analysis was applied to evaluate multiple prognostic factors.

**Results:** Tumor in non-upper lobe had worse OS (hazard ratio [HR]: 1.354, p < 0.001) and LCSS (HR: 1.476, p = 0.005) than the upper lobe in stage IB adenocarcinoma in 32-month landmark and IA3 (OS, HR: 1.300, p < 0.001; LCSS, HR: 1.413, p = 0.004) adenocarcinoma in 48-month landmark, but not in stage IA1 and IA2 adenocarcinoma. The results remained positive in subgroups of < 4, ≥ 4 and ≥ 11 LN examined in stage IB tumor and ≥ 4 LN examined in stage IA3 tumor. For SCC, non-upper lobar tumor had similar OS and LCSS with upper lobar tumor in all stages. The multivariate Cox analysis confirmed that the non-upper lobe was an independent risk factor in stage IA3-IB adenocarcinoma, but not in SCC. Adjuvant chemotherapy (ACT) could improve OS in stage IB adenocarcinoma (HR: 0.586, *p* < 0.001) and SCC (HR: 0.708, *p* = 0.030) located in non-upper lobe.

**Conclusions:** Non-upper lobar adenocarcinoma in stage IA3-IB was associated with worse prognosis. ACT may improve prognosis in stage IB tumor located in non-upper lobe.

## Introduction

Early stage lung and bronchus cancer accounts for about 17% of all diagnosed cases 1. According to the National Comprehensive Cancer Network (NCCN) guidelines, surgical resection plus lymph node (LN) dissection or sampling is recommended for stage I NSCLC and adjuvant chemotherapy (ACT) is only considered for high risk patients 2. The benefit of ACT for early stage NSCLC remains controversial. Identifying clinicopathologic features that may lead to worse prognosis may have clinical utility in offering adjuvant therapy to a subgroup of patients.

The correlation between tumor location and prognosis regarding operable NSCLC has been studied in previous studies 3. Most researches indicate that location matters in LN positive tumors 4, 5, and non-upper lobar tumor location is an adverse prognostic factor in stage III NSCLC 6, 7. Such differences in pathologic behavior may be attributed to stage migration 8-11, and non-upper lobe mainly drains into the subcarinal zone, which has significantly higher incidence of nodal upstaging 12. An alternative school of thought suggests that differences in ventilation and perfusion in the different zones are the main reason, leading to asymmetric distribution of aerosolized carcinogens, or other factors such as heterogeneity in angiogenesis 13, 14. For early stage NSCLC, the results diverge 15-17, and whether location matters in early stage NSCLC remains controversial. Some investigators conclude that stage migration, but not tumor location, is responsible for differences in survival between patients with upper and non-upper lobar tumors 17, 18. Considering that less LN examined may result in false negatives of nodal upstaging, whether location matters in stage I NSCLC adjusting for the LN examination should be evaluated. In addition, adenocarcinoma 19, 20 and higher T stage 21, 22 are also associated with higher incidence of nodal upstaging, so a comprehensive analysis should be performed.

In this study, we used the large Surveillance, Epidemiology, and End Results (SEER) database to explore the correlation between location and prognosis in stage I lung adenocarcinoma and squamous cell carcinoma (SCC), respectively, and performed subgroup analyses regarding LN examination.

## Patients and Methods

### Study population

This study was approved by the Institutional Review Board of Shanghai Pulmonary Hospital. Patients were selected from the SEER public use database, which contained data on cancer occurrences in 18 areas of the United States and covered approximately 34.6% of the population 23. Patients were included if the following inclusion criteria were met: (1) pathologically confirmed malignant primary stage I lung adenocarcinoma (ICD-0-3 code: 8140, 8141, 8143 or 8147, according to ICD-0-3 SEER Site/Histology Validation List) and SCC (ICD-0-3 code: 8070-8078) from 2004 to 2015; (2) History of lobectomy (surgery code: 33). The exclusion criteria were: (1) Primary tumor located in the main bronchus (primary site code: C34.0) or overlapping lesion of the lung (primary site code: C34.8), or unknown site (primary site code: C34.9); (2) receipt of radiation preoperatively, intraoperatively, or postoperatively, or if the radiation status was unknown.

All cases were restaged according to the 8^th^ edition of the lung cancer staging classification 24 by the tumor size. The baseline demographics of patients (age, sex, and race), characteristics of tumors (grade, site and laterality), treatment details (number of LN examined and chemotherapy), and outcomes (survival months, survival status and SEER cause-specific death classification) were collected by using the SEER*Stat program at May 03, 2020.

### Statistical analysis

**Figure 1:**
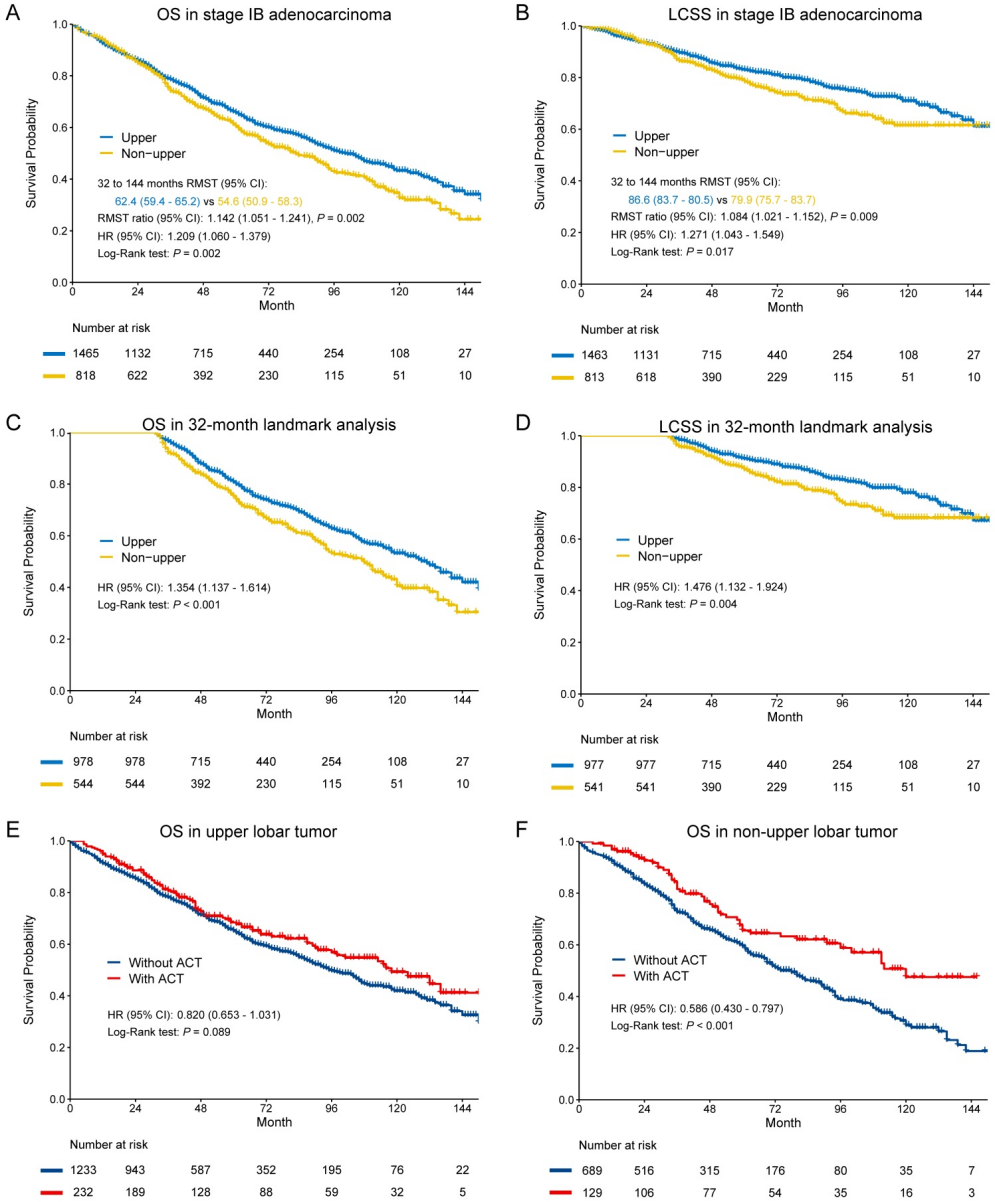
Kaplan-Meier survival estimates by location (upper vs. non-upper) for OS and LCSS in stage IB adenocarcinoma in the entire cohort (A-B) and 32-month landmark (C-D). Subgroup analyses regarding adjuvant chemotherapy in upper lobar (C) and non-upper (D) stage IB adenocarcinoma. Abbreviations: ACT, adjuvant chemotherapy; CI, confidence interval; HR, hazard ratio; LCSS, lung cancer-specific survival; OS, overall survival; RMST, restricted mean survival time.

Since patient's age was recorded in a 5-year interval, we defined the median of the interval as the age of the patient. For patients in the 85+ interval, 85 was regarded as the age. Categorical variables were analyzed by the Pearson chi-square test, and continuous variables were analyzed by the two-sample t test. Kaplan-Meier method was used to obtain the overall survival (OS) and lung cancer-specific survival (LCSS) of patients in the two groups and a log-rank test was used to compare the survival curves using the *survival* and *survminer* R package. The patients whose death reasons were unknown were excluded for LCSS analysis. As shown by the Kaplan-Meier survival curves in **Figure 1A-B and 3A-B**, survival curves of patients with non-upper lobar tumor declined synchronously with those of patients with upper lobar tumor during the first several months after surgery, then decreased more quickly. To better understand the association between tumor location and survival, restricted mean survival time (RMST) was calculated and compared to quantify long-term survival benefit using the *surv2sampleComp* R package, which was recommended by Horiguchi et al. 25. In addition, we also took 32 and 48 months as a landmark for stage IB and IA3 tumor in the survival analysis (**Figure 1C-D and 3C-D**). A full Cox proportional hazards model that included all the baseline variables was applied to adjust for candidate risk factors in the comparison and identify independent risk factors. The subgroup analyses regarding LN examination and ACT were conducted to explore its benefit in each group. Analyses were conducted using R software (version 3.6.3), and a two-sided *P* value of 0.05 was considered statistically significant.

## Results

Totally, 14399 patients with stage I lung adenocarcinoma and 7297 patients with stage I lung SCC were included. **Supplementary Table S1-3** listed the demographic and tumor characteristics of the entire patients with adenocarcinoma and SCC and the patients in landmark analysis, respectively.

### Stage IB

**Figure 2:**
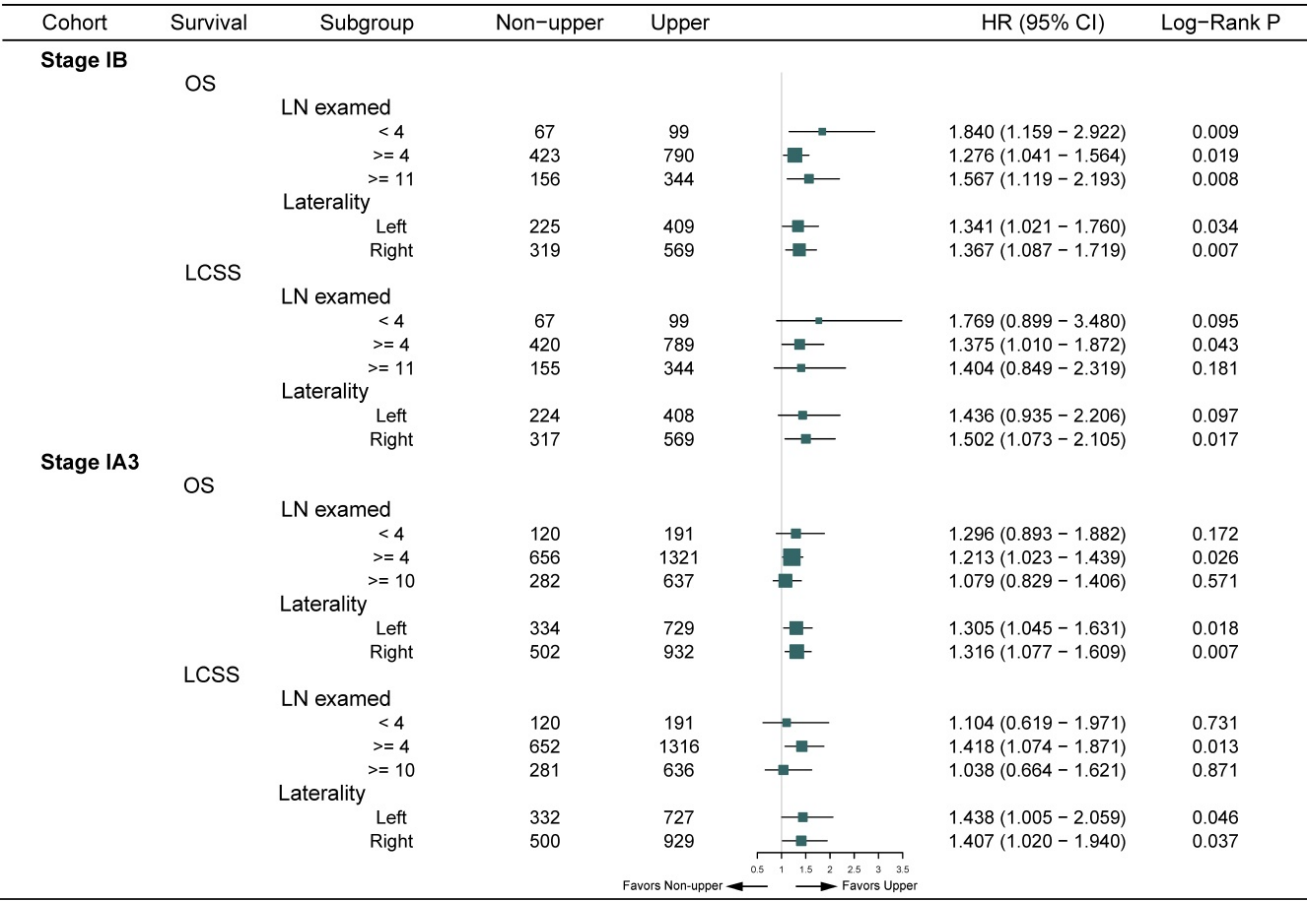
Subgroup analyses regarding LN examined and laterality of OS and LCSS in landmark. Abbreviations: CI, confidence interval; HR, hazard ratio; LCSS, lung cancer-specific survival; LN, lymph node; OS, overall survival.

In the entire cohort, non-upper lobar tumor was significantly associated with worse OS in adenocarcinoma (**Figure 1A-B**), and the multivariate Cox confirmed that it was an independent risk factor (**Supplementary Table S4**). Given that the Kaplan-Meier curves crossed between 0 and 32 months after surgery and then separated, we analyzed the RMST from 32 to 144 months. Non-upper lobar tumor had significant short RMST of OS (62.4 [59.4-65.2] vs 54.6 [50.9-58.3], ratio: 1.142 [1.051-1.241], *p* = 0.002, **Figure 1A**) and LCSS (86.6 [83.7-89.5] vs 79.9 [75.7-83.7], ratio: 1.084 [1.021-1.152], *p* = 0.009, **Figure 1B**). In addition, we took the patients with more than 32 months (**Supplementary Table S3**) in the following analyses. Non-upper lobar tumor had significantly worse OS (HR [95% CI]: 1.354 [1.137-1.614], *p* < 0.001) and LCSS (HR [95% CI]: 1.476 [1.132-1.924], *p* = 0.004) in 32-month landmark analysis (**Figure 1C-D**). The multivariate Cox analysis confirmed that tumor located in non-upper lobe was independently associated with worse OS (HR [95% CI]: 1.350 [1.132-1.611], *p* < 0.001) and LCSS (HR [95% CI]: 1.493 [1.143-1.949], *p* = 0.003) in adenocarcinoma (**Table 1**). To explore whether the potential false negatives of mediastinal metastasis was responsible for the differences, number of LN examined was stratified as < 4 and ≥ 4 and we also analyzed the patients with ≥ 11 LNs examined which was the optimal number for stage IB NSCLC proposed by Dai et al. 26. The results remained positive in the patients with ≥ 4 and ≥ 11 LNs examined (**Figure 2**), which indicated that potential stage migration due to less LN examined was not responsible for worse survival. We also performed the subgroup analysis regarding laterality, and the results were also positive (**Figure 2**).

The above results indicated that non-upper lobar tumor was a risk factor for survival, so we analyzed the benefit of ACT in the two groups. ACT could improve OS in non-upper lobar tumor (HR [95% CI]: 0.586 [0.430-0.797], *p* < 0.001,** Figure 1F**), but not in upper lobar tumor (HR [95% CI]: 0.820 [0.653-1.031], *p* = 0.089, **Figure 1E**). However, ACT could not improve LCSS in both upper (HR [95% CI]: 1.240 [0.914-1.683], *p* = 0.167) and non-upper (HR [95% CI]: 1.008 [0.687-1.479], *p* = 0.968) tumor (**Supplementary Figure S1A-B**).

**Figure 3:**
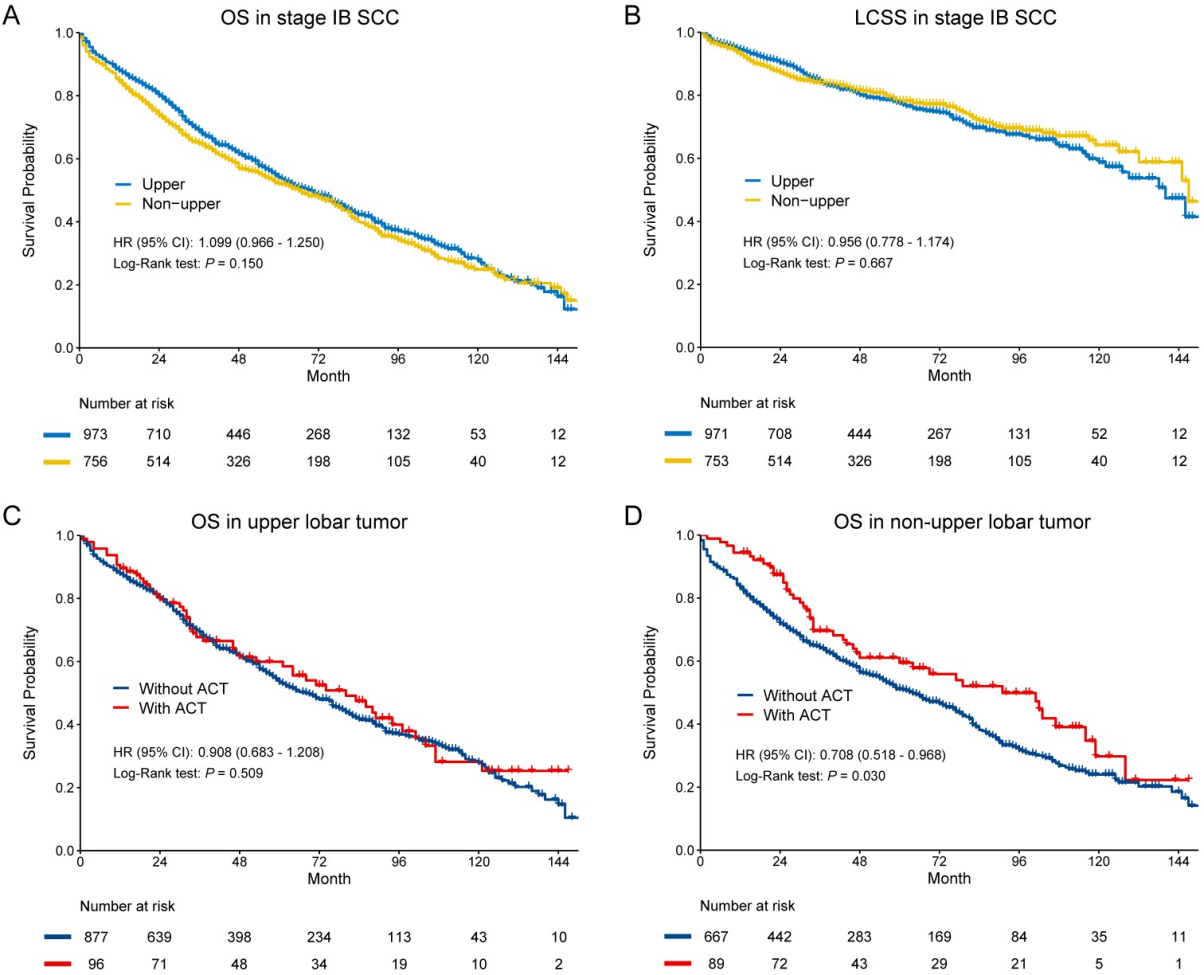
Kaplan-Meier survival estimates by location (upper vs. non-upper) for OS (A) and LCSS (B) in stage IB SCC. Subgroup analyses regarding adjuvant chemotherapy in upper lobar (C) and non-upper (D) stage IB SCC. Abbreviations: CI, confidence interval; HR, hazard ratio; LCSS, lung cancer-specific survival; OS, overall survival.

In SCC, there were no significant differences in OS (HR [95% CI]: 1.099 [0.966-1.250], *p* = 0.150) and LCSS (HR [95% CI]: 0.956 [0.778-1.174], *p* = 0.667) (**Figure 3A-B**). The multivariate Cox regression also showed that location was not the risk for OS (HR [95% CI]: 1.052 [0.923-1.199], *p* = 0.451) and LCSS OS (HR [95% CI]: 0.902 [0.746-1.134], *p* = 0.436) in SCC (**Table 2**). In subgroup analysis (**Figure 3C-D**), we observed that ACT could improve OS in non-upper lobar tumor (HR [95% CI]: 0.708 [0.518-0.968], *p* = 0.030), but not in upper lobar tumor (HR [95% CI]: 0.908 [0.683-1.208], *p* = 0.509). However, ACT could not improve LCSS in both upper (HR [95% CI]: 0.895 [0.576-1.392], *p* = 0.623) and non-upper (HR [95% CI]: 0.816 [0.499-1.334], *p* = 0.417) tumor (**Supplementary Figure S1C-D**).

### Stage IA3

**Figure 4:**
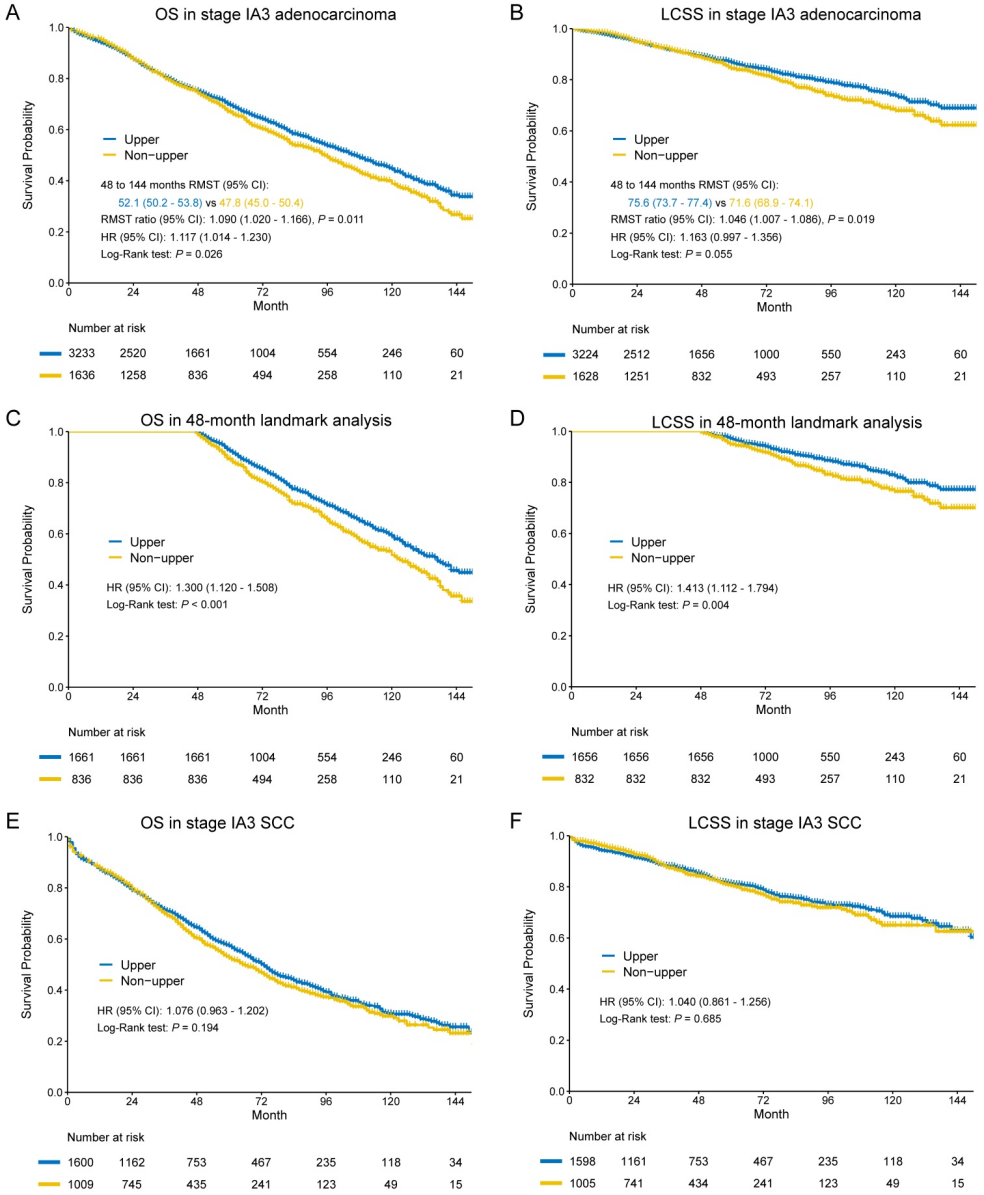
Kaplan-Meier survival estimates by location (upper vs. non-upper) for OS and LCSS in stage IA3 adenocarcinoma in the entire cohort (A-B) and 48-month landmark (C-D). Kaplan-Meier survival estimates by location (upper vs. non-upper) for OS and LCSS in stage IA3 SCC in the entire cohort (E-F). Abbreviations: CI, confidence interval; HR, hazard ratio; LCSS, lung cancer-specific survival; OS, overall survival; SCC, squamous cell carcinoma.

In the entire cohort, worse OS and LCSS were observed in non-upper lobar tumor in adenocarcinoma (**Figure 4A-B**), and the multivariate Cox regression also revealed the same result (**Supplementary Table S4**). The Kaplan-Meier curves crossed between 0 and 48 months, so we analyzed the RMST in the groups from 48 to 144 months. Non-upper lobar tumor had significant short RMST of OS (52.1 [50.2-53.8] vs 47.8 [45.0-50.8], ratio: 1.090 [1.020-1.166], *p* = 0.011, **Figure 4C**) and LCSS (75.6 [73.7-77.4] vs 71.6 [68.9-74.3], ratio: 1.046 [1.007-1.066], *p* = 0.019, **Figure 4D**). In addition, we analyzed the patients in 48-month landmark (**Supplementary Table S3**), and non-upper lobar tumor had significantly worse OS (HR [95% CI]: 1.300 [1.120-1.508], *p* < 0.001, **Figure 4C**) and LCSS (HR [95% CI]: 1.413 [1.112-1.794], *p* = 0.004, **Figure 4D**). The multivariate Cox analysis confirmed that tumor located in non-upper lobe was independently associated with worse OS (HR [95% CI]: 1.293 [1.112-1.502], *p* < 0.001) and LCSS (HR [95% CI]: 1.419 [1.113-1.807], *p* = 0.005) in adenocarcinoma (**Table 1**). We also performed subgroup analyses regarding the number of LN examined and laterality (**Figure 2**). The result was negative in the patients with ≥ 10 LNs examined (optimal number for stage IA3 26) and positive in the patients with ≥ 4 LNs examined, but not keeping positive in the patients with < 4 LNs examined, which demonstrated that stage migration probably was not responsible for the differences. Given that the sample size of the patients who received ACT was very small, subgroup analysis was not available.

In terms of SCC, no differences were observed in both OS (HR [95% CI]: 1.076 [0.963-1.202], *p* = 0.194) and LCSS (HR [95% CI]: 1.040 [0.861-1.256], *p* = 0.685) (**Figure 4E-F**). The results of multivariate Cox regression were consistent with Kaplan-Meier plots in both OS (HR [95% CI]: 1.016 [0.907-1.137], *p* = 0.786) and LCSS (HR [95% CI]: 0.989 [0.816-1.198], *p* = 0.908) (**Table 2**).

### Stage IA2

No survival differences were observed in both adenocarcinoma (OS: HR [95% CI]: 1.010 [0.920-1.110], *p* = 0.828; LCSS: HR [95% CI]: 0.977 [0.835-1.143], *p* = 0.770) and SCC (OS: HR [95% CI]: 1.090 [0.968-1.227], *p* = 0.153; LCSS: HR [95% CI]: 0.917 [0.726-1.157], *p* = 0.464) (**Supplementary Figure S2A-D**).

### Stage IA1

Location did not matter in both adenocarcinoma (OS: HR [95% CI]: 0.815 [0.635-1.048], *p* = 0.111; LCSS: HR [95% CI]: 0.659 [0.399-1.086], *p* = 0.102) and SCC (OS: HR [95% CI]: 0.979 [0.711-1.349], *p* = 0.899; LCSS: HR [95% CI]: 1.028 [0.553-1.902], *p* = 0.931) (**Supplementary Figure S3A-D**).

## Discussion

The correlation between tumor location and prognosis in early stage NSCLC was reported in previous studies. Ou et al. 15 and Wang et al. 27 reported two large population-based analyses of stage I NSCLC, and they observed that upper lobar tumor had significant better survival than non-upper lobar tumor for both stage IA and IB NSCLC. The results of 122 stage I NSCLC patients treated with stereotactic body radiation therapy (SBRT) reported by Shaverdian et al. 16 showed that the lower lobar tumor was associated with poor relapse-free and overall survival. However, Puri et al. 17 held the opposite view. A total of 621 patients with stage I NSCLC were included, and no significant difference between upper lobar tumor and other lobar tumor was observed in OS curve. All the three studies didn't perform subgroup analysis for specific histologic types and surgical procedures. Given that there were so many confounders in previous studies, we performed the study to give a more convincing result. We only included the patients who received lobectomy with mediastinal LN dissection, because lobectomy could bring better survival in stage I NSCLC 28, 29, and we divided the patients into upper and non-upper group due to its lymphatic drainage patterns.

In this study, we find that location mattered in stage IA3 and IB adenocarcinoma and patients with non-upper lobar tumor had significantly worse OS and LCSS, but not in SCC. However, the reason remained unclear and there were no robust verdicts. Stage migration was discussed in most studies. Non-upper lobe mainly drained into the subcarinal zone, and had higher incidence of metastasis. Liang at al. 10 analyzed N2 involvement in individual LN station and zones for specific lobes in 4511 patients. They found that the highest incidence of metastasis for RUL, RML, RLL, LUL and LLL were station 4, 7, 7, 5 and 7, respectively. For left lingular division, Haruki et al. 11 and Riquet et al. 30 both reported that the highest incidence of metastasis was upper zone, for which we did not combine RML with RUL as non-lower lobe to analyze in spite of the resembling anatomic location. Eckardt et al. 31 reported that unexpected subcarinal metastases were found in 5.9%, 5.1% and 1.6% of patients with a tumor in the lower, middle and upper lobe, respectively. Rocha et al. 12 also concluded that tumor located in a lower lobe was significantly associated with upstaging. We noticed that there were patients with < 4 LNs examined (lack of mediastinal LN probably), so we made subgroup analyses to explore the influence of potential false negatives of upstaging. If the results were positive in patients with less LN examined while negative in patients with more LN examined, stage migration was responsible for the differences probably. However, the results of subgroup analyses were not in accordance with the hypothesis, suggesting that potential stage migration may be not attributed to differences. The results preferred that heterogeneity in different location was the main reason, and the non-upper lolar tumor performed a greater likelihood of malignant behavior, which could not be reflected just by TNM stage. This was also the rationale of higher incidence of nodal upstaging in non-upper lobar tumor, with the same tumor size.

In terms of the difference between adenocarcinoma and SCC, the relatively weaker invasiveness of SCC may be the cause. Watanabe et al. 20 found that of all the patients with small size (2 cm or less) tumor who had mediastinal LN enlargement on chest CT, adenocarcinoma accounted for the major proportion (92.3%), while the proportion of SCC was 0%. Deng et al. 19, Libshitz et al. 32 and Kotoulas et al. 9 also reported that SCC was less likely to metastasize than adenocarcinoma, which reflected the weaker invasiveness of SCC. In stage IA1 and IA2 (≤ 2 cm) adenocarcinoma, the results indicated that location did not matter. Tumor size is a risk factor for mediastinal LN metastasis, and with the increasement of tumor size, the risk increased 21, 22. Overall, there may be nearly no large differences of malignant behavior in different locations in stage IA1-IA2 tumor and SCC, due to the weak invasiveness itself.

The choice of ACT for early stage NSCLC remains controversial. In this study, we explored the benefit of ACT in stage IB adenocarcinoma and SCC. We observed that chemotherapy could only improve OS in the patients with stage IB adenocarcinoma and SCC located in non-upper lobe, but not in upper lobe. As we mentioned above, non-upper lobar tumor may be associated with greater invasiveness and ACT could bring survival benefit in stage I tumor with greater invasiveness like micropapillary and solid subtypes of adenocarcinoma 33. However, the OS benefit of ACT was limited in our study, and we did not observe LCSS benefit and the small sample size of the patients who received ACT maybe account for it.

There were some limitations in this study. First, some biases were inevitable because of the retrospective nature of this study. Second, comorbidities, recurrence, central/peripheral location within the lobe, details of LN, details of lobectomy (sleeve or straightforward), performance status, pulmonary function, distribution of ventilation/perfusion across lobes for each individual patient were not available to make further analyses. Third, SEER database did not record the reason why the patients chose to receive ACT, and although not all the differentiation degrees of the patients who received ACT were poor, it was not clear whether there may be other risk factors. Forth, we just focused on the surgical population, and the patients who received SBRT or ablation were not available. Last, another independent validation cohort was lack in the study.

## Conclusion

This study demonstrated that non-upper lobar tumor was associated with worse survival in stage IA3-IB adenocarcinoma, but location did not matter in stage I SCC. ACT may improve prognosis in stage IB adenocarcinoma and SCC located in non-upper lobe. Future studies regarding early stage NSCLC should notice that location mattered in IA3-IB adenocarcinoma, and explore the benefit of ACT in different locations.

## Supplementary Material

Supplementary figures and tables.

## Figures and Tables

**Table 1:** Multivariate Cox analysis for OS and LCSS in patients with stage IA3-IB adenocarcinoma in 32- or 48-month landmark

	IA3 in 48-month landmark				IB in 32-month landmark			
	OS		LCSS		OS		LCSS	
	HR (95% CI)	*p*	HR (95% CI)	*p*	HR (95% CI)	*p*	HR (95% CI)	*p*
Age	1.043 (1.034-1.052)	<0.001	1.015 (1.002-1.029)	0.018	1.031 (1.021-1.041)	<0.001	0.991 (0.977-1.005)	0.191
**Race**								
White	1.000		1.000		1.000		1.000	
Black	1.121 (0.820-1.534)	0.473	0.926 (0.546-1.569)	0.774	1.128 (0.798-1.593)	0.495	0.741 (0.420-1.305)	0.298
Other	1.036 (0.778-1.379)	0.810	1.336 (0.879-2.030)	0.174	0.741 (0.528-1.041)	0.084	0.829 (0.502-1.371)	0.465
**Gender**								
Female	1.000		1.000		1.000		1.000	
Male	1.157 (0.999-1.339)	0.051	1.072 (0.845-1.360)	0.568	1.168 (0.981-1.392)	0.081	1.291 (0.991-1.682)	0.058
**Laterality**								
Left	1.000		1.000		1.000		1.000	
Right	0.854 (0.738-0.989)	0.034	0.866 (0.683-1.097)	0.233	0.958 (0.803-1.142)	0.633	1.026 (0.781-1.346)	0.856
**Differentiation grade**								
I	1.000		1.000		1.000		1.000	
II	1.591 (1.271-1.993)	<0.001	1.840 (1.249-2.710)	0.002	1.222 (0.941-1.588)	0.132	1.596 (1.024-2.488)	0.038
III	1.819 (1.428-2.316)	<0.001	2.046 (1.353-3.094)	<0.001	1.336 (1.014-1.760)	0.039	1.454 (0.910-2.322)	0.117
IV	0.383 (0.053-2.753)	0.340	1.111 (0.151-3.417)	0.917	1.279 (0.515-3.181)	0.596	1.155 (0.271-4.877)	0.846
**LN examed**								
< 4	1.000		1.000		1.000		1.000	
≥4	0.823 (0.671-1.009)	0.061	0.698 (0.509-0.958)	0.026	0.638 (0.496-0.820)	<0.001	0.601 (0.415-0.870)	0.007
Other †	0.696 (0.501-0.968)	0.031	0.743 (0.455-1.216)	0.238	0.749 (0.527-1.065)	0.107	0.683 (0.401-1.162)	0.160
**Chemotherapy**								
No	1.000 (reference)		1.000 (reference)		1.000 (reference)		1.000 (reference)	
Yes	0.992 (0.744-1.323)	0.958	1.211 (0.806-1.819)	0.356	0.874 (0.687-1.111)	0.270	1.151 (0.837-1.584)	0.386
**Location**								
Upper	1.000 (reference)		1.000 (reference)		1.000 (reference)		1.000 (reference)	
Non-upper	1.293 (1.112-1.502)	<0.001	1.419 (1.113-1.807)	0.005	1.350 (1.132-1.611)	<0.001	1.493 (1.143-1.949)	0.003

CI: confidence interval, HR: hazard ratio, LCSS: lung cancer-specific survival, LN: lymph node, OS: overall survival. † Number of nodes is unknown/not stated, or it is unknown whether nodes are examined.

**Table 2:** Multivariate Cox analysis for OS and LCSS in patients with stage IA3-IB SCC

	IA3				IB			
	OS		LCSS		OS		LCSS	
	HR (95% CI)	*p*	HR (95% CI)	*p*	HR (95% CI)	*p*	HR (95% CI)	*p*
Age	1.037 (1.029-1.044)	<0.001	1.018 (1.006-1.030)	0.003	1.038 (1.029-1.047)	<0.001	1.024 (1.011-1.038)	<0.001
**Race**								
White	1.000		1.000		1.000		1.000	
Black	0.917 (0.748-1.125)	0.407	0.958 (0.688-1.345)	0.802	0.771 (0.572-1.004)	0.054	0.692 (0.448-1.071)	0.098
Other	0.928 (0.691-1.248)	0.623	0.896 (0.534-1.503)	0.677	0.644 (0.444-0.933)	0.020	0.880 (0.531-1.456)	0.618
**Gender**								
Female	1.000		1.000		1.000		1.000	
Male	1.218 (1.090-1.362)	<0.001	1.199 (0.993-1.448)	0.060	1.307 (1.142-1.495)	0.001	1.206 (0.976-1.490)	0.083
**Laterality**								
Left	1.000		1.000		1.000		1.000	
Right	1.098 (0.984-1.226)	0.094	1.236 (1.024-1.493)	0.027	1.023 (0.898-1.165)	0.729	0.988 (0.805-1.214)	0.911
**Differentiation grade**								
I	1.000		1.000		1.000		1.000	
II	0.871 (0.622-1.219)	0.420	0.926 (0.518-1.656)	0.795	1.124 (0.787-1.605)	0.520	1.205 (0.669-2.170)	0.535
III	0.825 (0.589-1.157)	0.265	0.840 (0.468-1.506)	0.558	1.084 (0.759-1.548)	0.657	1.115 (0.619-2.010)	0.717
IV	0.897 (0.456-1.766)	0.754	0.781 (0.220-2.778)	0.703	1.220 (0.614-2.422)	0.571	1.135 (0.364-3.541)	0.827
**LN examed**								
< 4	1.000		1.000		1.000		1.000	
≥ 4	0.829 (0.708-0.971)	0.020	0.747 (0.577-0.967)	0.027	0.858 (0.703-1.047)	0.132	0.753 (0.557-1.016)	0.063
Other †	0.891 (0.702-1.131)	0.341	0.739 (0.490-1.115)	0.149	0.938 (0.716-1.228)	0.642	0.908 (0.603-1.366)	0.641
**Chemotherapy**								
No	1.000		1.000		1.000		1.000	
Yes	0.793 (0.586-1.074)	0.134	1.118 (0.783-1.802)	0.417	0.949 (0.765-1.177)	0.623	0.955 (0.683-1.336)	0.789
**Location**								
Upper	1.000		1.000		1.000		1.000	
Non-upper	1.016 (0.907-1.137)	0.786	0.989 (0.816-1.198)	0.908	1.052 (0.923-1.199)	0.451	0.902 (0.746-1.134)	0.436

CI: confidence interval, HR: hazard ratio, LCSS: lung cancer-specific survival, LN: lymph node, OS: overall survival. † Number of nodes is unknown/not stated, or it is unknown whether nodes are examined.
