# Supplementary material for: Correlation between tumor location and survival in stage I lung adenocarcinoma and squamous cell carcinoma: a SEER-based study
Source: J Cancer. 2021 Jun 22;12(17):5076–85. doi: 10.7150/jca.52572 (PMC8317522; doi:10.7150/jca.52572)
Supplement: Supplementary file 1 — Supplementary figures and tables. [file jcav12p5076s1.pdf]

Supplementary Tables

Table S1.

Demographic and tumor characteristics of patients with stage I adenocarcinoma.

	IA1			IA2			IA3			IB		
	Upper	Non-upper	*p*	Upper	Non-upper	*p*	Upper	Non-upper	*p*	Upper	Non-upper	*p*
N	690	373		4012	2172		3233	1636		1465	818	
Age, year (mean±SD)	65.1±9.0	64.5±9.6	0.712	66.3±9.4	67.4±9.2	<0.001	68.0±9.6	68.8±9.2	0.003	67.9±9.9	68.6±9.6	0.113
Race, %			0.779			0.676			0.057			0.725
White	590 (85.5)	319 (85.5)		3364 (83.8)	1811 (83.4)		2705 (83.7)	1367 (83.6)		1228 (83.8)	676 (82.6)	
Black	59 (8.6)	35 (9.4)		355 (8.8)	189 (8.7)		267 (8.3)	112 (6.8)		111 (7.6)	64 (7.8)	
Other	41 (5.9)	19 (5.1)		293 (7.3)	172 (7.9)		261 (8.0)	157 (9.6)		126 (8.6)	78 (9.4)	
Male gender, %	272 (39.4)	134 (35.9)	0.488	1737 (43.3)	821 (37.8)	<0.001	1399 (43.3)	677 (41.4)	0.219	662 (45.2)	339 (41.4)	0.092
Right-sided tumor, %	423 (61.3)	248 (66.5)	0.292	2439 (60.8)	1388 (63.9)	0.017	1889 (58.4)	989 (60.5)	0.185	840 (57.3)	499 (61.0)	0.097
Differentiation grade, %			0.163			<0.001			<0.001			0.012
I	207 (30.0)	138 (37.0)		815 (20.3)	541 (24.9)		566 (17.5)	358 (21.9)		223 (15.2)	132 (16.1)	
II	294 (42.6)	152 (40.8)		2112 (52.6)	1133 (52.2)		1610 (49.8)	835 (51.0)		653 (44.6)	414 (50.6)	
III	140 (20.3)	60 (16.1)		933 (23.3)	417 (19.2)		938 (29.0)	382 (23.3)		529 (36.1)	237 (29.0)	
IV	3 (0.4)	1 (0.3)		12 (0.3)	9 (0.4)		19 (0.6)	8 (0.5)		12 (0.8)	9 (1.1)	
NA	46 (6.7)	22 (5.9)		140 (3.5)	72 (3.3)		100 (3.1)	53 (3.2)		48 (3.3)	26 (3.2)	
LN examined, %			0.193			<0.001			0.001			0.037
< 4	89 (12.9)	63 (16.9)		488 (12.2)	360 (16.6)		373 (11.5)	247 (15.1)		150 (10.2)	111 (13.6)	
≥4	538 (78.0)	280 (75.1)		3199 (79.7)	1648 (75.9)		2589 (80.1)	1279 (78.2)		1194 (81.5)	633 (77.4)	
Other †	63 (9.1)	30 (8.0)		325 (8.1)	164 (7.6)		271 (8.4)	110 (6.7)		121 (8.3)	74 (9.0)	
Chemotherapy, %	18 (2.6)	12 (3.2)	0.706	161 (4.0)	68 (3.1)	0.092	181 (5.6)	108 (6.6)	0.182	232 (15.8)	129 (15.8)	1.000

LN: lymph node, NA: not available, SD: standard deviation.

† Number of nodes is unknown/not stated, or it is unknown whether nodes are examined.

Table S2.

Demographic and tumor characteristics of patients with stage I SCC.

	IA1			IA2			IA3			IB		
	Upper	Non-upper	*p*	Upper	Non-upper	*p*	Upper	Non-upper	*p*	Upper	Non-upper	*p*
N	299	119		1614	927		1600	1009		973	756	
Age, year (mean±SD)	68.9±8.4	69.7±7.5	0.382	69.3±8.1	69.8±7.8	0.178	69.4±8.3	70.9±8.2	<0.001	70.1±8.1	71.2±8.0	0.004
Race, %			0.710			0.360			0.059			0.439
White	270 (90.3)	106 (89.1)		1458 (90.3)	833 (89.9)		1387 (86.7)	898 (89.0)		845 (86.8)	669 (88.5)	
Black	19 (6.4)	7 (5.9)		114 (7.1)	61 (6.6)		157 (9.8)	72 (7.1)		86 (8.8)	54 (7.1)	
Other	10 (3.3)	6 (5.0)		42 (2.6))	33 (3.5)		56 (3.5)	39 (3.9)		42 (4.3)	33 (4.4)	
Male gender, %	147 (49.2)	65 (54.6)	0.369	885 (54.8)	477 (51.5)	0.109	982 (61.4)	538 (53.3)	<0.001	604 (62.1)	447 (59.1)	0.232
Right-sided tumor, %	177 (59.2)	73 (61.3)	0.769	887 (55.0)	573 (61.8)	0.001	829 (51.8)	626 (62.0)	<0.001	497 (51.1)	440 (58.2)	0.004
Differentiation grade, %			0.614			0.964			0.076			0.012
I	25 (8.4)	11 (9.2)		54 (3.3)	32 (3.5)		36 (2.2)	34 (3.4)		29 (3.0)	34 (4.5)	
II	160 (53.5)	67 (56.3)		887 (55.0)	500 (53.9)		764 (47.8)	513 (50.8)		435 (44.7)	367 (48.5)	
III	95 (31.8)	38 (31.9)		605 (37.5)	358 (38.6)		737 (46.1)	433 (42.9)		476 (48.9)	330 (43.7)	
IV	1 (0.3)	0 (0.0)		4 (0.2)	3 (0.3)		12 (0.8)	3 (0.3)		12 (1.2)	2 (0.3)	
NA	18 (6.0)	3 (2.5)		64 (4.0)	34 (3.7)		51 (3.2)	26 (2.6)		21 (2.2)	23 (3.0)	
LN examined, %			0.437			0.069			0.001			0.194
< 4	41 (13.7)	16 (13.4)		200 (12.4)	145 (15.6)		171 (10.7)	157 (15.6)		104 (10.7)	91 (12.0)	
≥4	232 (77.6)	97 (81.5)		1268 (78.6)	699 (75.4)		1306 (81.6)	777 (77.0)		791 (81.3)	589 (77.9)	
Other †	26 (8.7)	6 (5.0)		146 (9.0)	83 (9.0)		123 (7.7)	75 (7.4)		78 (8.0)	76 (10.1)	
Chemotherapy, %	13 (4.3)	5 (4.2)	1.000	38 (2.4)	32 (3.5)	0.133	69 (4.3)	35 (3.5)	0.332	96 (9.9)	89 (11.8)	0.233

LN: lymph node, NA: not available, SD: standard deviation.

† Number of nodes is unknown/not stated, or it is unknown whether nodes are examined.

Table S3.

Demographic and tumor characteristics of patients with stage IA3-IB adenocarcinoma with more than 32 or 48 months after surgery.

	IA3			IB		
	Upper	Non-upper	*p*	Upper	Non-upper	*p*
N	1661	836		978	544	
Age, year (mean±SD)	67.3±9.6	68.1±9.4	0.052	67.3±9.7	67.8±9.8	0.321
Race, %			0.019			0.543
White	1404 (84.5)	718 (85.9)		815 (83.3)	447 (82.2)	
Black	130 (7.8)	42 (5.0)		82 (8.4)	43 (7.9)	
Other	127 (7.6)	76 (9.1)		81 (8.3)	54 (9.9)	
Male gender, %	690 (41.5)	320 (38.3)	0.127	418 (42.7)	218 (40.1)	0.339
Right-sided tumor, %	932 (56.1)	502 (60.0)	0.067	569 (58.2)	319 (58.6)	0.904
Differentiation grade, %			0.001			0.038
I	286 (17.2)	182 (21.8)		164 (16.8)	94 (17.3)	
II	843 (50.8)	442 (52.9)		432 (44.2)	269 (49.4)	
III	472 (28.4)	191 (22.8)		344 (35.2)	154 (28.3)	
IV	13 (0.8)	1 (0.1)		7 (0.7)	9 (1.7)	
NA	47 (2.8)	20 (2.4)		31 (3.2)	18 (3.3)	
LN examined, %			0.054			0.334
< 4	191 (11.5)	120 (14.4)		99 (10.1)	67 (12.3)	
≥ 4	1321 (79.5)	656 (78.5)		790 (80.8)	423 (77.8)	
Other †	149 (9.0)	60 (7.2)		89 (9.1)	54 (9.9)	
Chemotherapy, %	102 (6.1)	66 (7.9)	0.117	169 (17.3)	97 (17.8)	0.841

LN: lymph node, NA: not available, SD: standard deviation.

Table S4.

Multivariate Cox analysis for OS and LCSS in patients with stage IA3-IB adenocarcinoma.

		IA3				IB			
		OS		LCSS		OS		LCSS	
		HR (95% CI)	*p*	HR (95% CI)	*p*	HR (95% CI)	*p*	HR (95% CI)	*p*
Age		1.037 (1.031 - 1.042)	<0.001	1.011 (1.003 - 1.019)	0.007	1.033 (1.025 - 1.041)	<0.001	1.004 (0.993 - 1.014)	0.471
Race									
	White	1.000		1.000		1.000		1.000	
	Black	1.163 (0.972 - 1.390)	0.099	1.112 (0.839 - 1.473)	0.461	1.084 (0.838 - 1.401)	0.540	0.912 (0.622- 1.337)	0.635
	Other	0.831 (0.685 - 1.008)	0.061	1.068 (0.805 - 1.418)	0.648	0.781 (0.608 - 1.003)	0.053	0.794 (0.542- 1.163)	0.256
Gender									
	Female	1.000		1.000		1.000		1.000	
	Male	1.373 (1.251 - 1.507)	<0.001	1.243 (1.071 - 1.442)	0.004	1.325 (1.163 - 1.508)	0.001	1.380 (1.136 - 1.678)	0.001
Laterality									
	Left	1.000		1.000		1.000		1.000	
	Right	0.987 (0.899 - 1.085)	0.790	1.081 (0.929 - 1.258)	0.315	1.022 (0.895 - 1.165)	0.750	1.129 (0.923 - 1.381)	0.236
Differentiation grade									
	I	1.000		1.000		1.000		1.000	
	II	1.462 (1.265 - 1.688)	<0.001	1.811 (1.402 - 2.340)	<0.001	1.518 (1.227 - 1.877)	<0.001	1.770 (1.249 - 2.509)	0.001
	III	1.916 (1.645 - 2.232)	<0.001	2.631 (2.018 - 3.429)	<0.001	1.797 (1.442 - 2.239)	<0.001	1.979 (1.382 - 1.835)	<0.001
	IV	1.291 (0.637 - 2.615)	0.479	3.018 (1.308 - 6.963)	0.010	1.313 (0.608 - 2.833)	0.488	1.247 (0.382 - 4.068)	0.714
LN examed									
	< 4	1.000		1.000		1.000		1.000	
	≥4	0.824 (0.742 - 0.938)	0.003	0.794 (0.648 - 0.974)	0.027	0.725 (0.600 - 0.875)	<0.001	0.716 (0.540 - 0.949)	0.020
	Other †	0.839 (0.687 - 1.025)	0.085	0.771 (0.559 - 1.063)	0.113	0.812 (0.620 - 1.064)	0.131	0.753 (0.499 - 1.135)	0.175
Chemotherapy									
	No	1.000 (reference)		1.000 (reference)		1.000 (reference)		1.000 (reference)	
	Yes	1.060 (0.878 - 1.280)	0.541	1.337 (1.033 - 1.731)	0.027	0.851 (0.705 - 1.027)	0.093	1.150 (0.899 - 1.472)	0.266
Location									
	Upper	1.000 (reference)		1.000 (reference)		1.000 (reference)		1.000 (reference)	
	Non-upper	1.128 (1.023 - 1.243)	0.015	1.193 (1.021 - 1.393)	0.026	1.214 (1.063 - 1.387)	0.004	1.292 (1.059 - 1.576)	0.011

CI: confidence interval, HR: hazard ratio, LCSS: lung cancer-specific survival, LN: lymph node, OS: overall survival.

† Number of nodes is unknown/not stated, or it is unknown whether nodes are examined.

Supplementary Figures

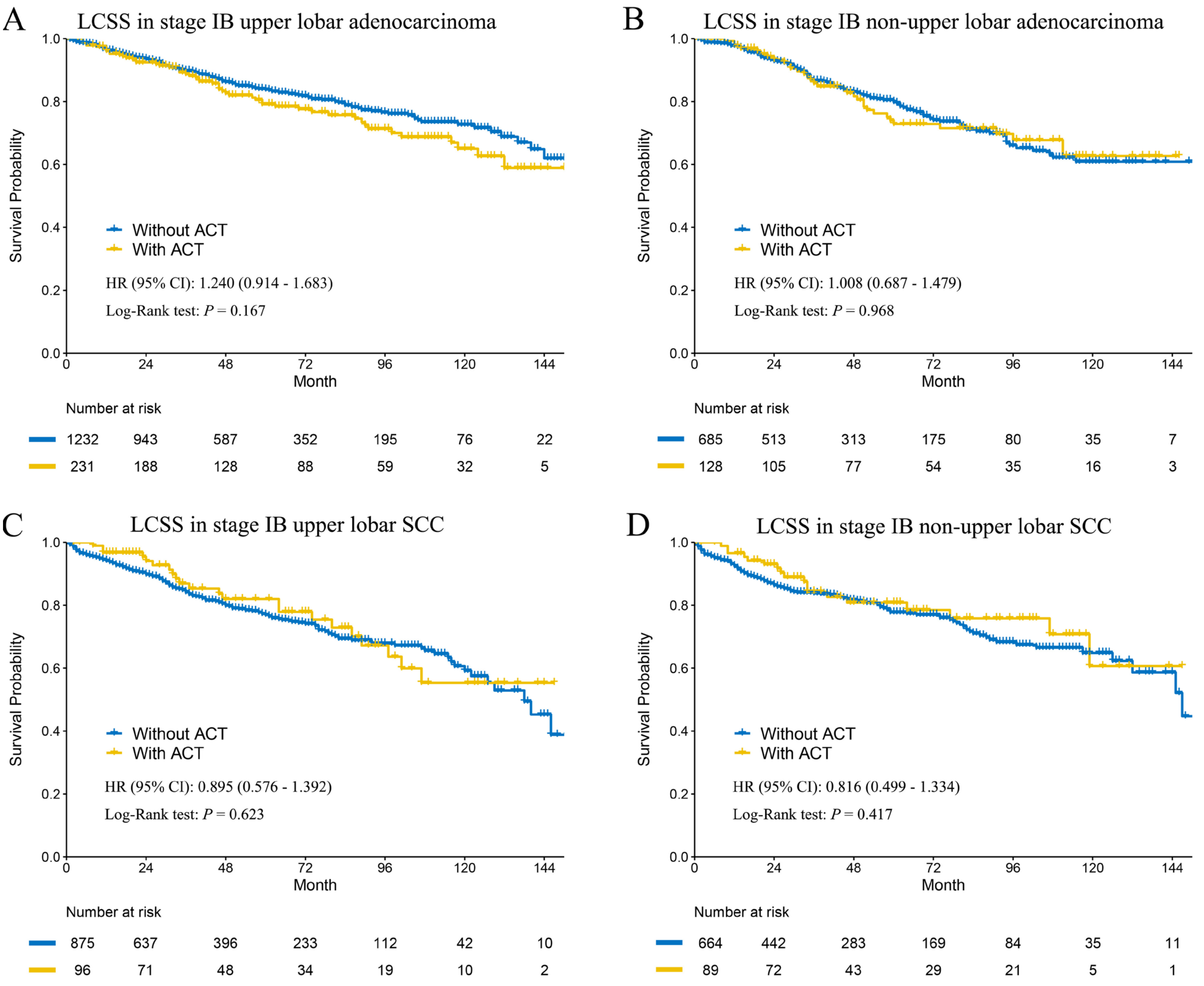

Figure S1.

Kaplan-Meier survival estimates by location (with ACT vs. without ACT) for LCSS in stage IB adenocarcinoma and SCC. (A) adenocarcinoma in upper lobe, (B) adenocarcinoma in non-upper lobe, (C) SCC in upper lobe, (D) SCC in non-upper lobe.

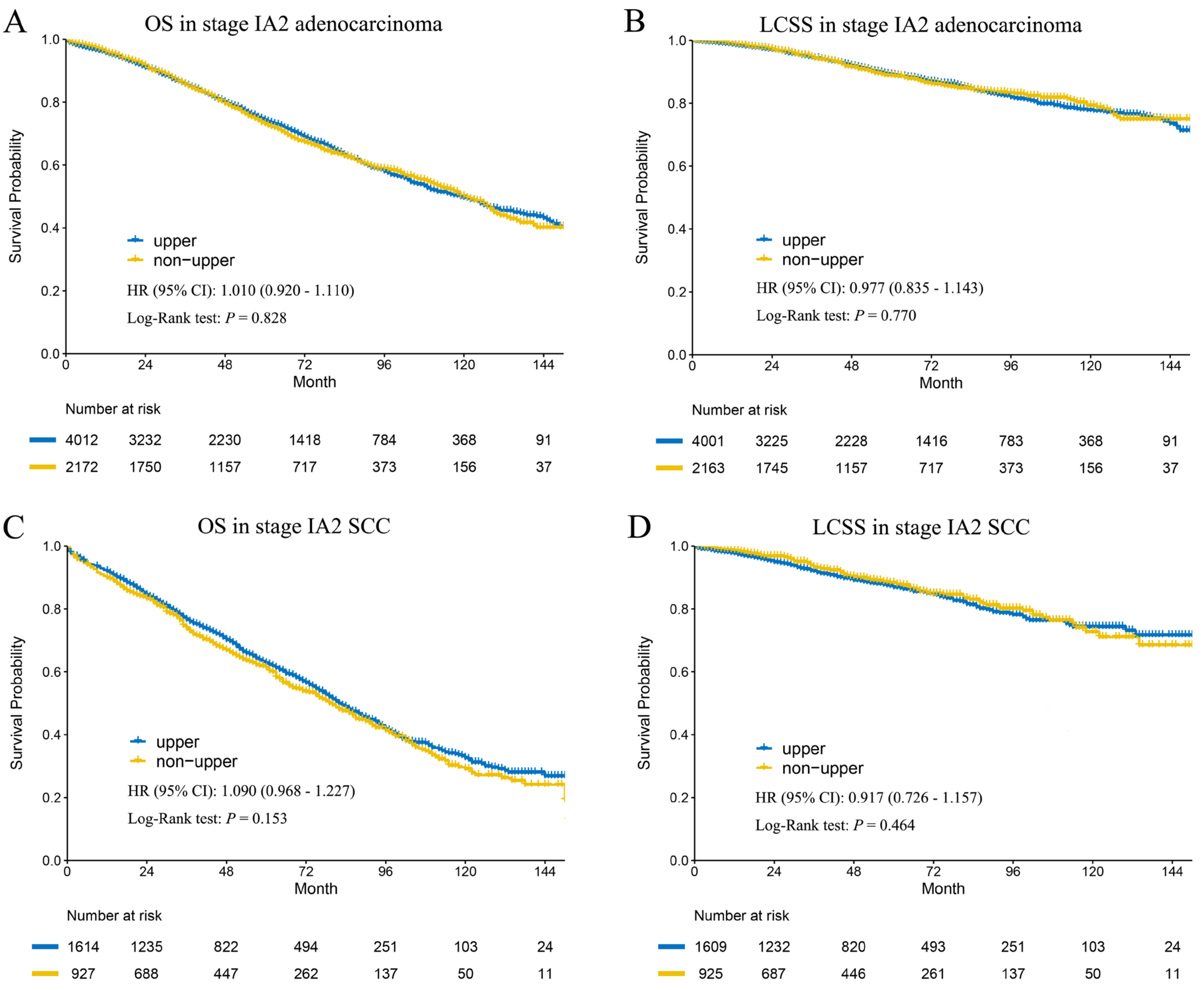

Figure S2.

Kaplan-Meier survival estimates by location (upper vs. non-upper) for OS and LCSS stage IA2 adenocarcinoma and SCC. (A) OS in adenocarcinoma, (B) LCSS in adenocarcinoma, (C) OS in SCC, (D) LCSS in SCC.

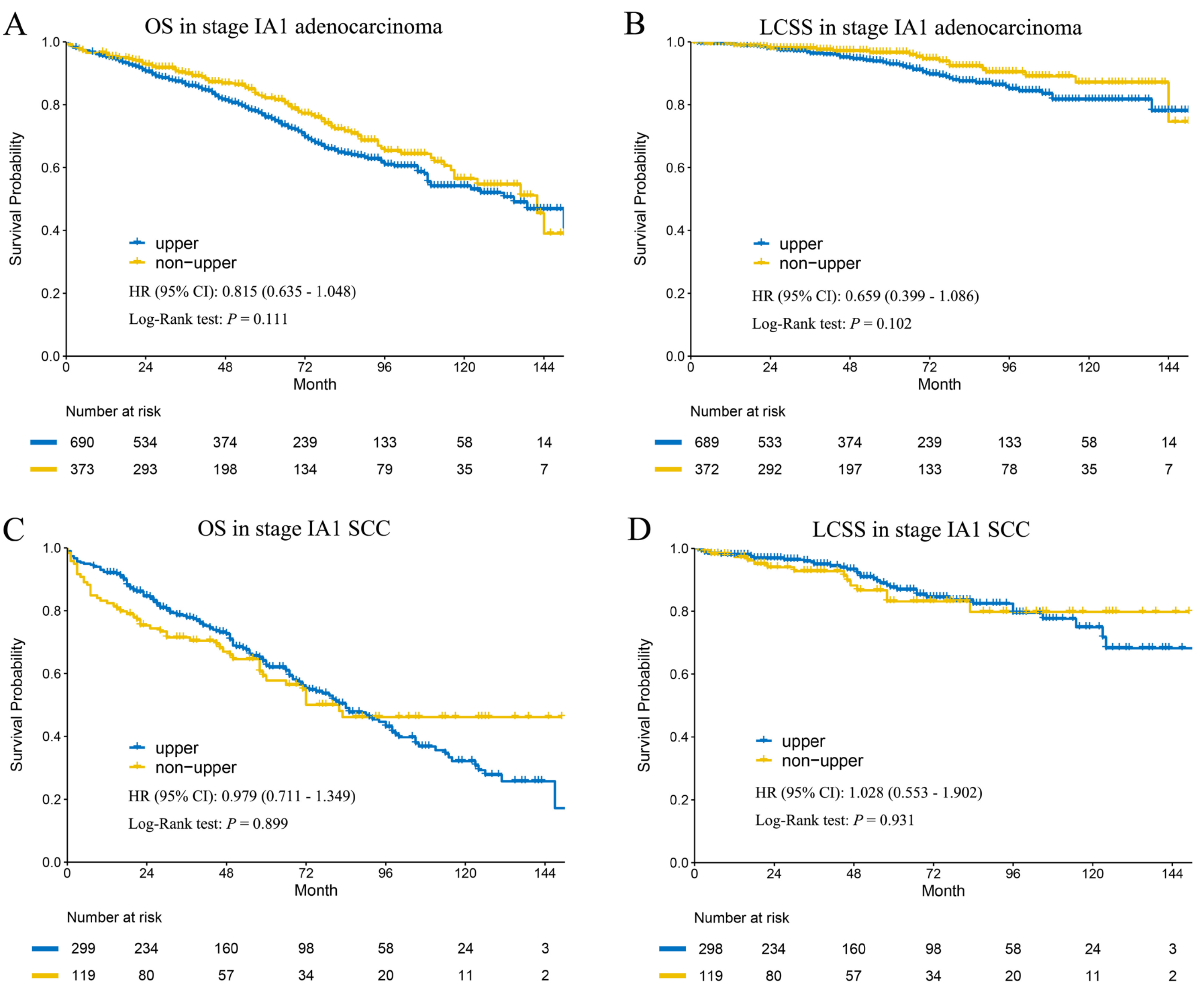

Figure S3.

Kaplan-Meier survival estimates by location (upper vs. non-upper) for OS and LCSS stage IA1 adenocarcinoma and SCC. (A) OS in adenocarcinoma, (B) LCSS in adenocarcinoma, (C) OS in SCC, (D) LCSS in SCC.
